# Jumping Motor Skills in Typically Developing Preschool Children Assessed Using a Battery of Tests

**DOI:** 10.3390/s24041344

**Published:** 2024-02-19

**Authors:** Ewa Gieysztor, Aleksandra Dawidziak, Mateusz Kowal, Małgorzata Paprocka-Borowicz

**Affiliations:** 1 Physiotherapy Department, Faculty of Health Sciences, Wroclaw Medical University, 50-367 Wrocław, Poland; aleksandra.dawidziak@student.umw.edu.pl (A.D.); mateusz.kowal@umw.edu.pl (M.K.); malgorzata.paprocka-borowicz@umw.edu.pl (M.P.-B.); 2 Scientific Club No. 15 Progressio Infantis, Physiotherapy Department, Faculty of Health Sciences, Wroclaw Medical University, 50-367 Wrocław, Poland

**Keywords:** jumping motor skills, preschool children, vertical jumps, wearable sensor, G-sensor, MOT test

## Abstract

The preschool period is characterised by the improvement in motor skills. One of the developmental tasks in children is the ability to jump. Jumping plays an important role in the development of leg strength and balance. It is the gateway to more complex movements. In the physiotherapy clinic, we see a lot of difficulties in jumping performance in 5–7-year-old children. The aim of this study is to present the jumping ability, assessed by the Motor Proficiency Test (MOT) and the G-sensor examination of the vertical countermovement jump (CMJ) and countermovement jump with arms thrust (CMJAT) parameters. A total of 47 children (14 boys and 33 girls) were randomly recruited. The mean age was 5.5 years. The mean height was 113 cm and the mean weight was 19.7 kg. The children were divided into two groups according to their results. Children with low basic motor skills have the greatest difficulty with jumping tasks. In the CMJ jump, the take-off force was lower than in the CMJAT (*p* = 0.04). Most CMJAT parameters correlate with age, weight, and height. Height correlates most with children’s jumping performance. This study may be useful for sport educators and developmental researchers. The topic should be further explored and the CMJ and CMJAT parameters may be established as a basis.

## 1. Introduction

The preschool period is characterised by the improvement in motor skills. One of the developmental tasks in children is the ability to jump. During development, the critical moment of obtaining the ability to jump is the age of 3 [1]. Jumping plays an important role in the development of leg strength and balance [2]. It requires the use of the upper and lower limbs and coordinated movements between them. Jumping is used in childhood play and games as well as in sport. It is the gateway to more specialised skills, more complex movements, and general physical activity [3]. However, in the physiotherapy clinic, we see a lot of difficulties in jumping performance in 5–7-year-old children, regarding both bipedal and single leg tests. The most difficult is jumping on one leg and changing the supporting leg. In addition, the inability to jump on each leg at the same level is often observed in preschool children. Testing children’s fundamental motor skills (FMSs) is popular during their development and many different tests are used for assessment [4,5,6,7,8,9,10]. The importance of physical development is enhanced because it is treated as information about intellectual and scholarly possibility [11]. The different levels of difficulties during neuromotor development involve a high number of children [7,12,13,14]. Therefore, monitoring the task possibilities is important to evaluate the level of child development. Some tests are used for the examination of motor and fine skills. They are usually informing about the level of the performance or the lack of possibility to do so. One of them is the Motor Proficiency Test (MOT 4–6), which evaluates five different jumping tasks among other tests. On the other hand, there are examinations focusing on specific skills, like a jump. There are many methods of examination jump tasks. Some of them measure vertical jumping, and some horizontal jumping or the mixed forms of jumping. One of the jump examinations is the vertical countermovement jump (CMJ) with and without arm movement, which is dedicated to measure neuromuscular performance [15,16,17]. The countermovement jump measures two parameters of the muscles—explosive strength and the elastic component [18].

The CMJ parameters have been widely used as an alternative measure of child development [19]. Studies show that children who perform the jump in its mature style have better results in jump height [20]. During the maturation process of the CMJ execution, there are three phases. It was found that six-year-old children perform an immature style of jumping and flex their knees less during the first phase; moreover, their jump is slower than in older children [21]. The age group of children that has been most studied is that of school-aged children. Some of the studies show that the CMJ is a very reliable test of neuromuscular performance in primary school children and indicate that there are no significant differences between the sexes in countermovement jump kinetics [22].

The aim of this study is to present the jumping ability of preschool children, assessed by two different tools, the Motor Proficiency Test (MOT) and the G-sensor examination, which provide the objective CMJ and CMJAT parameters. 

Some additional questions have been added:

What is the level of motor skills in preschool children?

Which tasks in the Motor Proficiency Test are the most difficult for children? 

Which tasks differentiate the groups of children with higher and lower motor skills measured by the MOT test? 

What are the kinetic parameters of the CMJ and CMJAT tests in preschool children?

As there is little research taking into account jumping ability in preschool children, the completion of data in this area seems to be anticipated.

## 2. Material and Methods

### 2.1. Participants 

A total of 47 children (14 boys and 33 girls) were randomly recruited from a kindergarten in Poland. The mean age of the group was 5.5 (±0.88) years. The mean height of the participants was 113 cm (±7.3) and the mean weight was 19.7 kg (±3.3). Inclusion criteria were typical development, understanding of instructions, and normal movement ability. Parents reported no pathological limb abnormalities at the time of testing nor any other musculoskeletal/neurological and/or cardiopulmonary condition likely to affect jumping ability. The exclusion criteria were physical or mental disability. All parents or guardians of the participants gave signed informed consent prior to the examination. This study was conducted in accordance with the Declaration of Helsinki, and the protocol was approved by the Ethics Committee of Wroclaw Medical University (Approval number KB-116/2019). 

Basic information about the participants is shown in Table 1.

### 2.2. Assessment of the Motor Proficiency

All data were collected in one session. The children’s motor skills were assessed using the Motor Proficiency Test (MOT). The test is most appropriate for this age group and consists of the most important tasks related to daily activities. The test consisted of 18 tasks [7,22,23]. They are divided into four categories: 1. Stability, 2. Locomotion, 3. Object control, 4. Fine motor skills. Tasks are to be performed by children. Five tasks are related to jumping. The detailed tasks are described in Table 2.

### 2.3. Assessment of the CMJ and CMJAT Jump Performance

The acquisition of the CMJ and CMJAT parameters was obtained using a BTS G-SENSOR measurement instrument (BTS Bioengineering Corp., Quincy, MA, USA). The device was equipped with a 16 bit/axis Triaxial Accelerometer with multiple sensitivities (±2, ±4, ±8, ±16 g), 16 bit/axis Triaxial Gyroscope with multiple sensitivities (±250, ±500, ±1000, ±2000°/s), and 13 bit Triaxial Magnetometer (±1200 uT). An inter-instrument correlation coefficient of between 0.90 and 0.99 and an intra-instrument coefficient of variation of ≤2.5% have demonstrated the suitability of the G-sensor for assessing physical activity [24,25,26].

All children were instructed to perform two kinds of jumps: a countermovement jump and countermovement jump with an arm thrust. The measurement of CMJ and CMJAT was carried out using the jump test protocol. The children were standing barefoot with their feet hip-width apart. The children made trial jumps and then performed jumps that were monitored with the G-sensor instrument, which was installed at the level of L5 in a belt designed for better attachment [18]. After a trial jump of each type, children performed one recorded CMJ and one CMJAT jump. Calibration was conducted prior to the assessment. There were one-minute breaks between jumps.

### 2.4. Statistics

A statistical analysis was performed using the TIBCO Statistica program (v. 13.3. 0, TIBCO Software Inc., Palo Alto, CA, USA; 2017). Arithmetic means and standard deviations were calculated for continuous variables. Normal distribution was assessed using the Kolmogorov–Smirnov test. A *t*-test was used for quantitative variables. Pearson’s r correlation analysis was used to determine the relationship between continuous variables and to determine the relationship between quantitative variables. The Mann–Whitney U test was used to compare two groups on ordinal variables. The level α ≤ 0.05 was used for comparisons.

## 3. Results

### 3.1. MOT Results

Children received 2 points for completing the more difficult version of the test, 1 point for completing the easier version of the task, and 0 points if the task was not completed.

The final scores were obtained by summing the points. The children were then divided into two groups according to their scores. Children with results below 25 points (lower level of physical fitness) were assigned to the first group (group 1), and those with the results from 25 points (higher level of physical fitness) were assigned to the second group (group 2).

The mean MOT result in group 1 was 21, and the mean MOT score in group 2 was 27. The difference was statistically significant (*p* < 0.05). With respect to ordinal variables, the Mann–Whitney U test was used.

The differences between groups in each MOT task are shown in Table 3.

During the analysis of the MOT results, we noticed that the greatest difference between the groups was in the tasks related to jumping ability. Taking this into account, we decided to carry out a more specific jumping assessment using the CMJ and CMJAT tests, in order to find out how the jumping parameters look in the preschool age group and whether the level of physical fitness is related to the CMJ and CMJAT performance and can be seen in the kinetic parameters.

### 3.2. CMJ and CMJAT Results for Two Groups of Children Divided by Fitness Ability

Comparing the parameters of two groups of children (group 1 and 2), there were no significant differences in CMJ and CMJAT results. Table 4 shows the results.

### 3.3. CMJ and CMJAT Results for the Whole Group of Children

During the CMJ and CMJAT, the following parameters were measured: maximum height (cm), maximum movement of the centre of mass (CoM), flight time (s), take-off force (kN), landing force (kN), velocity before flight (m/s), maximum velocity (m/s), mean velocity of the median phase (m/s), maximum concentric force (kW), mean concentric force (kW). The mean results of the group studied are shown in Table 5. All the parameters do not differ significantly between the two types of jumps, except for the take-off force, which was significantly lower in the CMJ jump performance (*p* = 0.04).

### 3.4. The Correlation between CMJ and CMJAT Results and Age, Weight, and Height

An analysis of the Pearson correlation coefficient (r) between age, weight, height, and CMJ and CMJAT parameters shows that most of the CMJAT parameters correlate with age, weight, and height. Seven out of ten are correlated with these parameters (see Table 6). Height correlates most with children’s jumping performance.

Moreover, we conducted the analysis of correlation. No strong correlations were found between CMJ and CMJAT parameters and jumping tasks in the MOT test. 

There were also no significant differences between girls and boys when analysing gender differences in CMJ and CMJAT parameters. The results are shown in the Supplementary Material in Tables S1 and S2.

## 4. Discussion

The objective of this study was to present the jumping ability of preschool children, assessed by two different tools, the Motor Proficiency Test (MOT) and the G-sensor examination, which provide the CMJ and CMJAT parameters. Moreover, some questions have been asked: What is the level of motor skills in preschool children? Which tasks in the Motor Proficiency Test are the most difficult for children? Which tasks differentiate the groups of children with higher and lower motor skills measured by the MOT test? What are the kinetic parameters of the CMJ and CMJAT tests in preschool children?

Analysing the results, we understood that a preschool child is in the process of development. There is not much basic information about the obligatory movement achievements, especially with regard to jumping. However, children’s motor ability is a common aspect observed to monitor proper development [27]. Jumping is one of the most popular tests, which is also the natural movement that is recreated in many movement games and popular play [28]. In the preschool period, this ability is still developing but is expected to be achieved [29]. This research, focused on preschool children’s ability to jump, shows the different tests used to measure this task.

In order to answer the question about the level of motor skills in preschool children and to look for the most difficult tasks for the children, which differentiate the groups of children with higher and lower motor skills, the MOT results were analysed. They showed the divergence between the developmental levels of children in the same age group. These differences were significant and the most difficult tasks for the children tested were definitely jumping tasks. Clinically, there is also a big problem with jumping in children [30]. In our opinion, this is due to fewer opportunities for natural movements during play. Nowadays, children are more often restricted by parents and teachers and they spend less time in spontaneous play [28]. There is even a tendency to engage preschool children in activity through special programmes of physical activity in order to improve their capacity for fitness [12,30]. 

Considering the problems with jumping, a more specific study was carried out. Vertical jumps (VJs) were analysed: countermovement jumps with and without arm movement.

CMJ and CMJAT are the popular forms of measuring elastic muscle components and explosive strength. The jumps differ in the use of the hands. The CMJ starts from an upright position with hands on hips, followed by a downward movement and immediately a vertical jump. The CMJAT starts with the same body position. The hands are at the sides and are extended upwards during the test [18,31]. The results of this study suggest that there is no statistically significant difference between the parameters of these two types of jumps. The only parameter that significantly differed was the take-off force. The result was higher for the CMJAT. The VJs are usually used to characterise the parameters of adults or athletes [32,33,34,35,36,37,38]. Children’s measurements are rare, but in the literature we found that Koren et al. used these tests involving 4-to-6-year-olds who were assessed longitudinally. They show almost the same average results in this group of children as in ours [39]. 

After analysing the effect of age, weight, and height on jumping performance in the groups studied, we found that the children did not differ significantly in age and weight, but they did differ in height. The children who were taller had better results in the MOT tests specific to jumping ability. These parameters were found to be interdependent with four parameters in the CMJ and seven parameters in the CMJAT. Koren et al. found that CMJ height progresses significantly with age [39]. In our results, the mean concentric force for CMJ was higher with age. For CMJAT, the results highlighted maximum height, flight time, landing force, and maximum and mean concentric force.

We did not observe any significant differences between the sexes in the kinetics of the countermovement jumps. The same result was found by Jones et al. in school-aged children and by Koren et al. in preschool children [22,39].

Jumping ability has also been shown to be a predictive test for neuromuscular diseases such as Charcot–Marie–Tooth disease. Therefore, the screening of children by jumping tests can extend the physical examination, which can monitor the health status of children [28,40]. We can observe it in relation to lower neuromaturity based on active primitive reflexes in preschool and school-aged children [23,41].

In order to analyse the motor skills of preschool children, we have used two assessment methods. The results of the MOT test show that children with lower physical performance have significantly lower jump test results. Surprisingly, these differences were not visible in the CMJ and CMJAT parameters. The difference in these two tests’ results may be related to different forms of jumping performance in the MOT test and the VJ. 

The practical issue of this study can be useful for clinicians, sports coaches, sports educators, as well as development researchers, especially those who analyse movement parameters.

## 5. Limitations

This study has some limitations. It is an observational study, so the first may be the lack of an intervention to compare the results before and after. In addition, this study should be extended by using different research equipment to analyse other parameters of vertical or horizontal jumping in children of this age group. A spontaneous movement analysis can be taken into account to observe the child’s behaviour.

## 6. Conclusions

Children with low basic motor skills have the greatest difficulty with jumping tasks. The parameters of vertical jumps correlate with age, weight, and height. For a better understanding of the jumping ability in preschool children, the data of the annual levels of jumping performance should be collected. This will make it possible to compare developmental periods. As this is a new topic, more research should be carried out and the CMJ and CMJAT parameters should be used as a basis.

## Figures and Tables

**Table 1:** Age, weight, and height description of the group.

	Age (y)	Weight (kg)	Height (cm)
M	5	19.68	113.41
(±)	0.88	3.32	7.26
min	3	14.80	98.00
max	7	27.60	128.00

M—mean, (±)—standard deviation, min—minimum, max—maximum, y—year, kg—kilograms, cm—centimetres.

**Table 2:** MOT 4–6 test—the items’ description [23].

1. Forward jump in a hoop	7. Carrying balls from box to box	13. Catching a tennis ring
2. Forward balance	8. Reverse balance	14. Jumping jacks
3. Placing dots on a sheet	9. Throwing at a target disk	15. Jumping over a cord
4. Grasping a tissue with toes	10. Collecting matches	16. Rolling around the long axis of the body
5. Sideward jump	11. Passing through a hoop	17. Standing up holding a ball on the head
6. Catching a stick	12. Jumping in a hoop on 1 foot, standing on 1 leg	18. Jump and turn in a hoop

**Table 3:** The comparison of group 1 and 2 in the MOT tasks.

	Rang Sum Group 1	Rang Sum Group 2	U	Z	*p*
Forward jump in a hoop	610.00	425.00	204.00	−0.78	0.43
Jumping in a hoop on 1 foot	516.00	519.00	110.00	−2.98 *	0.001 *
Jump and turn in a hoop	615.50	419.50	209.50	−0.66	0.51
Forward balance	533.50	501.50	127.50	−2.58	0.01 *
Reverse balance	579.00	456.00	173.00	−1.51	0.13
Grasping a tissue with toes	605.00	430.00	199.00	−0.90	0.37
Catching a stick	618.00	417.00	212.00	−0.60	0.55
Carrying balls from box to box	624.00	411.00	218.00	−0.46	0.65
Throwing at a target disk	590.00	445.00	184.00	−1.25	0.21
Catching a tennis ring	498.00	537.00	92.00	−3.41	0.001 *
Jumping jacks	548.50	486.50	142.50	−2.22	0.03 *
Jumping over a cord	509.00	526.00	103.00	−3.15	0.001 *
Sideward jump	491.50	543.50	85.50	−3.56	0.001 *
Rolling around the long axis of the body	590.50	444.50	184.50	−1.24	0.21
Standing up holding a ball on the head	638.50	396.50	232.50	−0.12	0.91
Collecting matches	586.00	449.00	180.00	−1.35	0.18
Placing dots on a sheet	584.50	450.50	178.50	−1.38	0.17
Sum of points, MOT 4–6	406.00	629.00	0.00	−5.56	0.001 *

* statistically significant difference; U—the U-value represents the number of times observations in one sample precede observations in the other sample in the ranking; Z—a number representing how many standard deviations above or below the mean population the score derived from a z-test is; *p*—*p*-value.

**Table 4:** CMJ and CMJAT results for two groups of children divided by fitness ability.

	CMJ Mean ±			CMJAT Mean ±		
	Group 1	Group 2	*p*-Value	Group 1	Group 2	*p*-Value
Maximum Height (cm)	11.84 (±3.53)	11.41 (±4.79)	0.76	10.45 (±3.50)	12.09 (±4.91)	0.25
Max Movement of the CoM	16.89 (±5.28)	17.05 (±5.65)	0.93	15.83 (±5.07)	18.12 (±5.90)	0.22
Flight Time (s)	0.31 (±0.05)	0.29 (±0.08)	0.58	0.29 (±0.05)	0.30 (±0.08)	0.41
Take-Off Force (kN)	0.16 (±0.06)	0.18 (±0.10)	0.50	0.20 (±0.08)	0.22 (±0.08)	0.55
Landing Force (kN)	0.34 (±0.13)	0.34 (±0.17)	0.99	0.36 (±0.12)	0.42 (±0.12)	0.16
Velocity before Flight (m/s)	1.72 (±0.33)	1.62 (±0.47)	0.50	1.62 (±0.39)	1.75 (±0.48)	0.40
Max Velocity (m/s)	1.85 (±0.33)	1.78 (±0.44)	0.63	1.75 (±0.37)	1.89 (±0.48)	0.37
Mean Velocity of the Median Phase (m/s)	0.88 (±0.28)	1.05 (±0.27)	0.08	0.90 (±0.25)	1.00 (±0.33)	0.31
Maximum Concentric Force (kW)	0.54 (±0.15)	0.57 (±0.25)	0.65	0.55 (±0.19)	0.64 (±0.21)	0.18
Mean Concentric Force (kW)	0.22 (±0.08)	0.27 (±0.11)	0.10	0.23 (±0.09)	0.28 (±0.10)	0.17

CMJ—countermovement jump; CMJAT—countermovement jump with arm thrust; cm—centimetre; CoM—centre of mass; s—second; kN—kiloNewton; m/s—metre per second; kW—kilowatt.

**Table 5:** CMJ and CMJAT results for the whole group of children.

	CMJ Mean ±	CMJAT Mean ±	*p*-Value
Maximum Height (cm)	11.68 (±3.98)	11.09 (±4.12)	0.54
Max Movement of the CoM	16.95 (±5.34)	16.72 (±5.45)	0.86
Flight Time (s)	0.30 (±0.06)	0.29 (±0.06)	0.55
Take-Off Force (kN)	0.17 (±0.07)	0.21 (±0.08)	0.04 *
Landing Force (kN)	0.34 (±0.14)	0.38 (±0.12)	0.18
Velocity before Flight (m/s)	1.68 (±0.39)	1.67 (±0.42)	0.92
Max Velocity (m/s)	1.82 (±0.37)	1.81 (±0.41)	0.86
Mean Velocity of the Median Phase (m/s)	0.95 (±0.28)	0.94 (±0.28)	0.90
Maximum Concentric Force (kW)	0.55 (±0.19)	0.59 (±0.20)	0.45
Mean Concentric Force (kW)	0.24 (±0.01)	0.25 (±0.01)	0.64

* statistically significant difference; CMJ—countermovement jump; CMJAT—countermovement jump with arm thrust; cm—centimetre; CoM—centre of mass; s—second; kN—kiloNewton; m/s—metre per second; kW—kilowatt.

**Table 6:** The correlation between CMJ and CMJAT results and age, weight, and height.

	CMJ			CMJAT		
	Age	Weight	Height	Age	Weight	Height
Maximum Height (cm)	0.20	0.16	0.16	0.36 *	0.27	0.50 *
Max Movement of the Centre of Mass (CoM)	0.11	0.19	0.13	0.32	0.21	0.46 *
Flight Time (s)	0.22	0.21	0.12	0.35 *	0.26	0.48 *
Take-Off Force (kN)	0.28	0.57 *	0.33 *	0.31	0.22	0.22
Landing Force (kN)	0.23	0.53	0.21	0.50 *	0.50 *	0.47 *
Velocity before Flight (m/s)	0.17	0.22	0.08	0.18	0.12	0.31
Max Velocity (m/s)	0.17	0.22	0.09	0.22	0.13	0.32
Mean Velocity of the Median Phase (m/s)	0.17	0.18	0.07	0.25	0.19	0.35
Maximum Concentric Force (kW)	0.32	0.68 *	0.46 *	0.47 *	0.56 *	0.64 *
Mean Concentric Force (kW)	0.35	0.62 *	0.38 *	0.42 *	0.51 *	0.59 *

* *p* < 0.05; CMJ—countermovement jump; CMJAT—countermovement jump with arm thrust; cm—centimetre; CoM—centre of mass; s—second; kN—kiloNewton; m/s—metre per second; kW—kilowatt.

## Data Availability

Data are contained within the article and Supplementary Materials.

## Supplementary Materials

The following supporting information can be downloaded at: https://www.mdpi.com/article/10.3390/s24041344/s1, Table S1: Correlation between MOT test results and CMJ and CMJAT results. Table S2: Differences in test results of CMJ and CMJAT between boys and girls.
