# Supplementary material for: Jumping Motor Skills in Typically Developing Preschool Children Assessed Using a Battery of Tests
Source: Sensors (Basel). 2024 Feb 19;24(4):1344. doi: 10.3390/s24041344 (PMC10893251; doi:10.3390/s24041344)
Supplement: Supplementary file 1 [file sensors-24-01344-s001.zip › sensors-2847206-supplementary.pdf]

Table S7. Correlation between MOT test results and CMJ and CMJAT results.

	Countermovement jump (CMJ)									
	Maximum Height (cm)	Max Movement of the CoM	Flight Time (s)	Take-Off Force (kN)	Landing Force (kN)	Velocity before Flight (m/s)	Max Velocity (m/s)	Mean Velocity of the Median Phase (m/s)	Maximum Concentric Force (kW)	Mean Concentric Force (kW)
Forward jump in a hoop	-0,34	-0,32	-0,30	0,02	-0,07	-0,26	-0,27	-0,28	-0,18	-0,25
Jumping in a hoop on 1 foot	0,17	0,26	0,13	0,04	0,00	0,13	0,15	0,14	0,09	0,06
Jump and turn in a hoop	0,16	0,09	0,10	-0,10	-0,05	0,02	0,04	0,12	-0,12	-0,02
Forward balance	0,28	0,32	0,21	0,05	-0,05	0,19	0,22	0,29	0,08	0,15
Reverse balance	0,29	0,16	0,21	0,04	-0,13	0,13	0,15	0,24	0,02	0,14
Grasping a tissue with toes	0,38	0,30	0,38	0,12	0,17	0,28	0,29	0,44	0,19	0,35
Catching a stick	-0,11	-0,10	-0,18	-0,38	-0,10	-0,14	-0,14	-0,17	-0,05	-0,22
Carrying balls from box to box	0,14	0,24	0,16	0,19	0,25	0,28	0,31	0,22	0,25	0,24
Throwing at a target disk	0,19	0,34	0,21	0,18	0,22	0,21	0,24	0,24	0,31	0,27
Catching a tennis ring	0,36	0,29	0,35	0,25	0,25	0,31	0,31	0,35	0,32	0,34
Jumping Jacks	-0,19	-0,21	-0,23	-0,23	-0,28	-0,30	-0,29	0,11	-0,28	0,01
Jumping over a cord	0,23	0,15	0,23	0,31	0,12	0,17	0,18	0,20	0,28	0,30
Sideward jump	0,14	0,14	0,10	0,07	0,01	0,03	0,06	0,03	0,15	0,10
Rolling around the long axis of the body	0,07	-0,01	0,06	0,19	-0,16	-0,02	0,00	-0,07	0,02	-0,03
Standing up holding a ball on the head	0,02	0,06	-0,02	-0,27	-0,30	-0,10	-0,07	-0,18	-0,32	-0,40
Collecting matches	-0,04	-0,02	-0,03	0,05	0,07	0,03	0,04	-0,16	0,22	-0,01
Placing dots on a sheet	0,01	-0,02	-0,01	0,08	0,21	-0,04	-0,02	0,17	0,00	0,13
Sum points MOT 4-6	0,23	0,14	0,18	-0,18	-0,20	0,06	0,09	0,21	-0,17	0,00

	Countermovement jump with arms thrust (CMJAT)									
	Maximum Height (cm)	Max Movement of the CoM	Flight Time (s)	Take-Off Force (kN)	Landing Force (kN)	Velocity before Flight (m/s)	Max Velocity (m/s)	Mean Velocity of the Median Phase (m/s)	Maximum Concentric Force (kW)	Mean Concentric Force (kW)
Forward jump in a hoop	-0,17	-0,17	-0,17	-0,09	0,03	-0,11	-0,08	0,02	-0,20	-0,10
Jumping in a hoop on 1 foot	0,13	0,21	0,12	0,00	0,13	0,07	0,06	0,06	0,06	0,07
Jump and turn in a hoop	0,01	0,11	-0,03	0,01	0,21	-0,02	0,02	0,05	-0,09	-0,05
Forward balance	0,06	0,11	0,01	0,13	0,20	0,02	0,03	0,09	-0,02	0,08
Reverse balance	0,27	0,14	0,24	0,17	0,27	0,23	0,26	0,38	0,20	0,34
Grasping a tissue with toes	0,11	0,06	0,06	0,29	0,30	0,06	0,08	0,09	0,19	0,16
Catching a stick	0,05	0,07	0,06	-0,15	0,20	-0,07	-0,07	0,06	-0,05	0,06
Carrying balls from box to box	0,28	0,25	0,28	0,34	0,46	0,29	0,30	0,49	0,31	0,50
Throwing at a target disk	0,14	0,25	0,14	0,02	-0,08	0,09	0,05	-0,16	0,16	-0,08
Catching a tennis ring	0,17	0,16	0,13	0,17	0,18	0,14	0,17	0,11	0,24	0,18
Jumping Jacks	-0,13	-0,06	-0,16	-0,10	-0,13	-0,16	-0,17	-0,05	-0,17	-0,11
Jumping over a cord	0,24	0,31	0,23	0,13	0,32	0,24	0,26	0,29	0,28	0,35
Sideward jump	0,34	0,26	0,32	0,15	0,31	0,20	0,20	0,15	0,32	0,25
Rolling around the long axis of the body	0,04	-0,03	-0,03	0,13	0,03	-0,01	0,01	0,13	0,02	0,16
Standing up holding a ball on the head	-0,16	-0,19	-0,17	-0,04	-0,31	-0,11	-0,12	-0,20	-0,31	-0,37
Collecting matches	0,31	0,32	0,29	0,17	0,35	0,19	0,22	0,30	0,28	0,30
Placing dots on a sheet	0,03	-0,06	0,00	0,20	0,09	-0,04	-0,04	0,11	0,04	0,17
Sum points MOT 4-6	0,24	0,27	0,18	0,22	0,23	0,19	0,22	0,21	0,15	0,16

CMJ - countermovement jump; CMJAT - countermovement jump with arm thrust; cm - centimetre; CoM - center of mass; s - second; kN - kiloNewton; m/s - metre per second; kW – kilowatt

Table S8. Differences in test results of CMJ and CMJAT between boys and girls.

	CMJ			CMJAT		
	**Boys**	**Girls**	**p-value**	**Boys**	**Girls**	**p-value**
Maximum Height (cm)	11.38	11.86	0.74	11.45	10.88	0.70
Max Movement of the CoM	16.58	17.17	0.76	17.48	16.30	0.54
Flight Time (s)	0.30	0.30	0.99	0.30	0.29	0.53
Take-Off Force (kN)	0.18	0.16	0.57	0.20	0.21	0.82
Landing Force (kN)	0.38	0.32	0.23	0.38	0.38	0.90
Velocity before Flight (m/s)	1.70	1.67	0.87	1.74	1.63	0.48
Max Velocity (m/s)	1.83	1.82	0.94	1.87	1.77	0.49
Mean Velocity of the Median Phase (m/s)	0.85	1.00	0.15	0.94	0.94	0.95
Maximum Concentric Force (kW)	0.59	0.53	0.39	0.62	0.57	0.43
Mean Concentric Force (kW)	0.22	0.25	0.50	0.25	0.25	0.82

CMJ - countermovement jump; CMJAT - countermovement jump with arm thrust; cm - centimetre; CoM - center of mass; s - second; kN - kiloNewton; m/s - metre per second; kW – kilowatt
